# Tryptophan and Its Metabolite Serotonin Impact Metabolic and Mental Disorders via the Brain–Gut–Microbiome Axis: A Focus on Sex Differences

**DOI:** 10.3390/cells14050384

**Published:** 2025-03-06

**Authors:** Mengyang Xu, Ethan Y. Zhou, Haifei Shi

**Affiliations:** 1 Program in Cell, Molecular, and Structural Biology, Miami University, Oxford, OH 45056, USA; 2 Institute for the Environment and Sustainability, Miami University, Oxford, OH 45056, USA

**Keywords:** gut microbiome, brain–gut–microbiome axis, tryptophan, serotonin, obesity, eating disorder, affective disorder, sex difference, estrogen

## Abstract

The crisis of metabolic and mental disorders continues to escalate worldwide. A growing body of research highlights the influence of tryptophan and its metabolites, such as serotonin, beyond their traditional roles in neural signaling. Serotonin acts as a key neurotransmitter within the brain–gut–microbiome axis, a critical bidirectional communication network affecting both metabolism and behavior. Emerging evidence suggests that the gut microbiome regulates brain function and behavior, particularly through microbial influences on tryptophan metabolism and the serotonergic system, both of which are essential for normal functioning. Additionally, sex differences exist in multiple aspects of serotonin-mediated modulation within the brain–gut–microbiome axis, affecting feeding and affective behaviors. This review summarizes the current knowledge from human and animal studies on the influence of tryptophan and its metabolite serotonin on metabolic and behavioral regulation involving the brain and gut microbiome, with a focus on sex differences and the role of sex hormones. We speculate that gut-derived tryptophan and serotonin play essential roles in the pathophysiology that modifies neural circuits, potentially contributing to eating and affective disorders. We propose the gut microbiome as an appealing therapeutic target for metabolic and affective disorders, emphasizing the importance of understanding sex differences in metabolic and behavioral regulation influenced by the brain–gut–microbiome axis. The therapeutic targeting of the gut microbiota and its metabolites may offer a viable strategy for treating serotonin-related disorders, such as eating and affective disorders, with potential differences in treatment efficacy between men and women. This review would promote research on sex differences in metabolic and behavioral regulation impacted by the brain–gut–microbiome axis.

## 1. Introduction

### 1.1. Overview of Tryptophan Metabolism

Tryptophan is not only an essential amino acid for protein synthesis but also a precursor for many important bioactive compounds. Besides conversion to tryptamine, three metabolic pathways process tryptophan to produce (i) serotonin (5-hydroxytryptamine, 5-HT) and melatonin, (ii) kynurenine, and (iii) indole acetic acid. All three pathways occur in both the central nervous system (CNS) and the peripheral enteric nervous system (ENS), allowing tryptophan metabolites to extensively regulate a wide range of metabolic and psychiatric functions crucial to health [1]. Numerous associations between tryptophan metabolites and various diseases have been documented in the literature. This review focuses on 5-HT, a key CNS neurotransmitter that plays a critical role in numerous physiological processes, including mood regulation, feeding behavior, and energy metabolism.

A detailed description of tryptophan metabolism and the synthesis of its metabolites [2], however, is beyond the scope of this review. Additionally, tryptophan metabolites from the kynurenine and indole acetic acid pathways are known to be important metabolic regulators involved in inflammation and immune responses [2], but these are not the focus of this review.

### 1.2. Overview of the Brain–Gut–Microbiome Axis

#### 1.2.1. Microbiome

The brain–gut–microbiome axis is a complex, bidirectional communication network that enables continuous crosstalk among the brain, gut, resident gut microbiota, and microbial metabolites. This communication occurs through a combination of neural and circulating signals [3,4,5]. Exciting emerging research suggests that gut microbial influence plays a fundamental role in regulating behavior and metabolism, with the brain–gut–microbiome axis being essential for maintaining homeostasis under both healthy and disease conditions [5,6,7,8,9,10,11].

**Figure 1:**
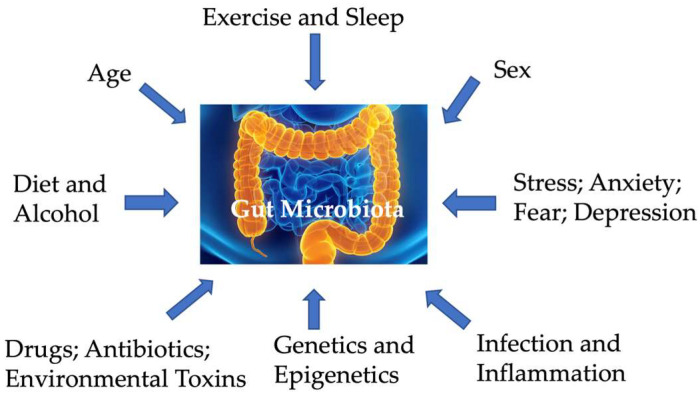
Factors impacting gut microbiota.

The microbiome consists of all microorganisms residing in and on the body, including fungi, protozoa, archaea, viruses, and bacteria, with the gastrointestinal tract hosting the largest microbial collection. The gut microbiome encompasses all these microorganisms that reside within the gut, their genetic repertoire (including number, classes, variability, and diversity of genes), and the metabolites they produce. Acting symbiotically with the host, the gut microbiome ferments dietary fibers, aids in digestion and absorption, and protects against pathogens. The gut microbiome is influenced by multiple factors, including the host’s genetics, age, and sex [12,13,14,15,16,17,18,19,20]; lifestyle (such as diet, alcohol, exercise, and sleep (e.g., [21])); and chronic diseases (Figure 1). The gut microbiota is also heterogeneous across the gastrointestinal tract, with varying bacterial communities from the oral region to the distal colon, as well as between the mucosal and the luminal layer [22,23]. Microbial composition changes over different life stages within individuals, especially during key transitional periods of brain development and maturation. In early life, microbial composition and metabolites change in parallel to meet the dynamic metabolic demands of growing brains [24]. Moreover, diet significantly impacts gut microbiota composition, which affects food digestion, subsequent energy harvest, and immune development-related epithelial health and homeostasis [25]. For instance, although *Firmicutes*, *Bacteroidetes*, *Actinobacteria*, and *Verrucomicrobia* are typical phyla in healthy adult microbiota, the ratio of *Firmicutes* to *Bacteroidetes* (i.e., *Firmicutes/Bacteroidetes)* varies widely across individuals [26,27].

With the spread of the Western diet, obesity incidence has been increasing worldwide over the past few decades. Obesity is a key risk factor for low-grade chronic inflammation, which is a likely contributing factor to various complications, including colorectal cancer, as well as type 2 diabetes and cardiovascular disease [28]. Markedly different microbiota profiles exist between lean and obese individuals [29,30]. Manipulating gut microbiota composition with probiotics and prebiotics may improve weight management and fat deposition. High-fat diet (HFD) feeding has been shown to induce epigenomic changes, including histone modifications, in both humans and animal models [31]. Understanding how the host gut microbiome and epigenome respond to diet could provide mechanistic insights into diseases associated with obesity. For instance, probiotic supplementation with *Lactobacillus* and *Bifidobacterium* in an animal model of HFD-fed mice has led to reduced weight gain and better metabolic outcomes, such as improved glucose homeostasis and insulin sensitivity [32]. Clinical studies suggest that microbiota modifications, such as receiving gut microbiota from lean donors, may ameliorate symptoms associated with obesity and improve insulin sensitivity in individuals with metabolic syndrome [33]. Therefore, the interaction between gut microbiota and hosts involves a wide range of pathways and presents a promising avenue that affects health and disease.

#### 1.2.2. Communication Using Neural and Circulating Signals

**Figure 2:**
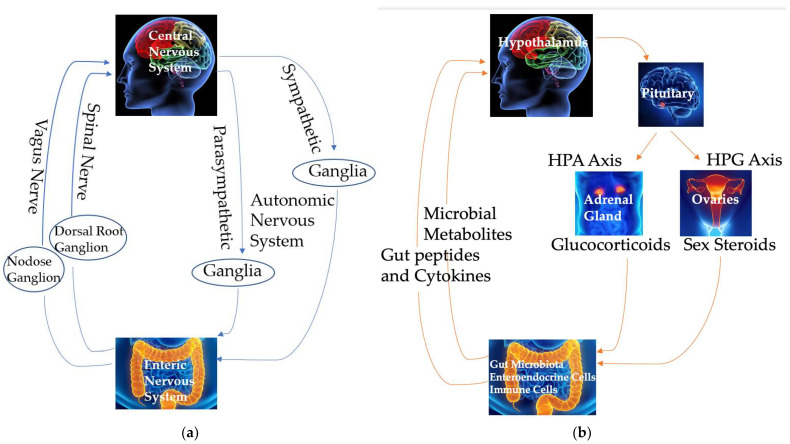
Communication using neural (**a**) and circulating (**b**) signals in the brain–gut–microbiome axis. HPA: hypothalamic–pituitary–adrenal. HPG: hypothalamic–pituitary–gonadal.

The brain significantly influences gut motility, permeability, secretion, immune functions, gut microbial gene expression, and the structure and function of the microbiome. This interplay between the brain and the gastrointestinal tract relies on both neural and circulating signals to modulate metabolic and immune functions [34,35,36] (Figure 2).

##### Neural Signals

The ENS, a complex neural network within the gut, facilitates bidirectional communication in the brain–gut axis [37]. The ENS consists of three anatomical ganglionated plexuses of submucosal, myenteric, and subserous plexuses, interconnected by fibers [36]. Five major types of enteric neurons, based on their morphological, neurochemical, and functional characteristics, are intrinsic primary afferent neurons, interneurons, and excitatory, inhibitory, and secreto-motor neurons [38,39]. These neurons form a bidirectional network, with afferent neurons in the submucosal and myenteric plexuses responding to chemical and mechanical stimuli and regulating vasodilation and blood flow, motility, mucosal transport, secretion, and nutrient absorption [40]. While the ENS operates autonomously to some extent, it is ultimately integrated with local reflexes and neural connections involving the autonomic nervous system and vagal and spinal afferents connecting the gut and the CNS, and also interacts with the gut immune and endocrine systems to regulate various gastrointestinal functions [39,41] (Figure 2a).

Efferent neural signals

Efferent parasympathetic vagal signals, relayed through the dorsal motor nucleus of the vagus (DMV), control gut motility and secretion [37]. Efferent sympathetic signals originate from neurons in the intermediolateral tract (IML) of the spinal cord. These signals travel via splanchnic and pelvic spinal pathways, synapse in the prevertebral ganglia, and influence vagal connections and enteric neurons, thereby inhibiting both gut secretion and motility [42] (Figure 2a).

Afferent neural signals

Communication between the gut microbiota and the host is complex and not fully understood, but evidence suggests that gut microbes can activate afferent sensory neurons. For example, the probiotic *Lactobacillus reuteri* enhances afferent excitability, modulating gut motility and pain perception [43]. Changes in microbial composition and metabolomics have been linked to alterations in neurotransmitter expression within both the CNS and ENS, which are crucial for controlling gut sensory-motor functions [44].

Afferent neural pathways convey signals from the gut to the brain via vagal and spinal sensory inputs. The soma of the vagal afferents resides in the nodose vagal ganglion and transmits sensory information about gastrointestinal motor activity and distension due to food presence to the nucleus of the solitary tract (NTS) in the brainstem [37]. Similarly, afferent spinal neurons, located in the dorsal root ganglia, relay signals from the gut to secondary afferent neurons in the dorsal horn. These secondary neurons project to the CNS via spinothalamic pathways, serving as major pain signaling routes [37] (Figure 2a). These vagal and spinal inputs are directed to higher brain regions, including the prefrontal cortex, amygdala, hypothalamus, emotional motor system, and limbic system, thereby participating in long vago–vagal reflexes [37]. Additionally, sensory signals from the gut to the brain help regulate reflex activity and mood states [5].

##### Circulating Signals

Efferent circulating signals

Some hormones regulating the functions of the brain–gut–microbiome axis and 5-HT synthesis and metabolism are produced by the hypothalamic–pituitary–adrenal (HPA) axis and the hypothalamic–pituitary–gonad (HPG) axis (Figure 2b). The activation of these axes begins with the release of corticotropin-releasing hormone (CRH) from the hypothalamus, stimulating the anterior pituitary gland to secrete adrenocorticotropic hormone (ACTH). This, in turn, prompts the adrenal cortex to release glucocorticoids, primarily cortisol in humans. The HPA axis plays a crucial role in mediating the stress response and also influences visceral sensation, gut motility and permeability, and immune functions, particularly during stress. These effects can subsequently affect emotional and cognitive functions in the brain [45] and influence the brain–gut–microbiome axis. This review will focus on the impact of sex steroid hormones from the HPG axis, particularly estrogen, on tryptophan metabolites, especially 5-HT, and the brain–gut–microbiome axis. In this review, the term “sex hormone”, instead of “gonadal hormone” and “reproductive hormone”, is used, as many organs other than the gonads produce estrogens and the effects of sex hormones discussed in this review are beyond their reproductive regulation.

Afferent circulating signals

Enteroendocrine cells, located in the epithelial lining of the gastrointestinal tract, are crucial for communication between the gut and the brain. In mammals, including humans, the most abundant type of enteroendocrine cell in the colon and rectum is enterochromaffin cells. These cells sense luminal constituents, such as food components, nutrients, bacterial toxins [46,47], and microbial metabolites like short-chain fatty acids (SCFAs; Figure 2b) [48]. The basolateral surface of these enteroendocrine cells interacts with both afferent and efferent nerves in the lamina propria [49,50,51]. Various sensory receptors on the apical surface of enteroendocrine cells detect luminal constituents, modulating the secretion of gut peptides and active biological molecules [52,53] and modulating local and systemic neural networks and functions [54,55].

The gastrointestinal epithelium represents the largest endocrine system in the human body [56], containing over 15 types of enteroendocrine cells that release more than 20 signaling molecules and hormones, including 5-HT, somatostatin, neuropeptide Y, and cholecystokinin, in response to diverse physiological and pathological stimuli [57,58,59,60] (Figure 2b). Receptors for many of these signaling molecules are located along the brain–gut axis, including in the ENS, on vagal afferents, and in the CNS [61,62,63], enabling effective communication between the gut and brain [64]. This brain–gut communication not only regulates gastrointestinal functions (e.g., gastric motility and acid secretion) but also influences brain functions (e.g., metabolic and behavioral responses) [50,51]. The impact of the gut microbiome and tryptophan metabolites on behavior highlights the potential role of gut alterations in the pathophysiology of metabolic and psychiatric disorders regulated by the CNS.

#### 1.2.3. Experimental Strategies in Tryptophan Metabolite and Gut Microbiome Research

Using a metagenomic approach, it has been estimated that the gut microbiome contains microorganisms at levels 10-fold greater than all the cells in the human body and has genes that are 150 times more abundant than the human genome [65,66,67]. This vast genetic diversity of the gut microbiome offers a wide range of metabolic, nutritional, hormonal, neural, and immune signals for crosstalk between the host and the gut. These signals directly and indirectly affect local metabolite production, control metabolic functions, regulate immune responses, and modulate behaviors, thus mutually achieving the maintenance of host homeostasis [68,69].

Several experimental strategies are available to researchers in this field. First, changes in tryptophan levels through depletion, supplementation, or molecular manipulation, either acutely or chronically, modify the peripheral and central levels of tryptophan metabolites. For example, preclinical and clinical studies employing these procedures have reported that low levels of the tryptophan metabolite 5-HT are associated with dysregulated eating behavior in animal models and eating disorders in humans, establishing the role of 5-HT in regulating feeding within the CNS. Second, accumulating evidence from animal models manipulating gut microbiota composition—through germ-free animals devoid of microorganisms, antibiotic-treated animals with microbiota deficiency, or animals rich in microorganisms via probiotic supplementation, fecal transplantation, or deliberate infections—suggests that the gut microbiome significantly impacts behavior and metabolism in lab animals, contributing to psychiatric and metabolic disorders seen in humans [11].

These strategies, through rigorous assessment using specific pathogen-free mice and germ-free mice, have illuminated the integrity of the gut microbiota in the functions of tryptophan metabolites, enhanced our understanding of the intricate relationship between the gut microbiome and the brain, and highlighted the crucial role of microbial communities in mediating various functions, including neural activity, metabolism, and behavior [70,71,72].

### 1.3. Aim and Focus of This Review

Research on tryptophan metabolites in health and disease is an active field. The PubMed database was searched for studies published up to November 2024 using a combination of keywords, including “tryptophan metabolism OR tryptophan metabolites OR tryptophan”, “estrogen OR sex hormone OR sex difference”, “brain–gut–microbiome axis OR gut microbiome OR gut microbiota”, “nervous system OR central nervous system OR peripheral nervous system OR enteric nervous system”, and “diseases OR disorders”. The returned hits were further screened based on the inclusion criteria of if serotonin was the focus of the studies and if metabolic and mental disorders and related physiology and behavior were studied. Most publications in the available literature, however, either do not link the influence of tryptophan and serotonin in metabolism with their influence in metabolic and behavioral regulation (e.g., [21]), or do not focus on sex differences and sex hormones on metabolic and mental health (e.g., [73]).

This review emphasizes the roles of the tryptophan metabolite 5-HT as a crucial player in brain–gut–microbiome crosstalk. The gut microbiome significantly impacts host health [74], influencing both regionally on nutrient digestion and absorption and systemically on whole-body metabolism and behavior [35]. Disruptions in gut microbiota composition and metabolites are associated with the pathophysiology of metabolic disorders such as diabetes and obesity, as well as neurologic and psychiatric diseases including eating disorders and affective disorders, such as anxiety and depression [75].

Notably, sex differences have been observed in tryptophan metabolism, as well as in the concentration and availability of tryptophan metabolites within the brain–gut–microbiome axis (Section 2); such findings, however, were rarely linked to sex differences in the regulation of physiology and behavior and related metabolic and mental disorders. Although many tryptophan metabolites may carry out sex-specific effects on the brain–gut–microbiome axis to influence health, we focus on the functional roles of 5-HT in influencing the CNS and regulating behaviors related to anxiety and depression (Section 3). Understanding sex differences in the effects of 5-HT on the brain–gut–microbiome axis is crucial for creating prevention and treatment strategies for metabolic, mental, and eating disorders, which should be specifically tailored to address the distinct needs of men and women.

In this review, we evaluate the existing evidence supporting the brain–gut–microbiome axis’s influence on tryptophan metabolism; review preclinical animal studies and clinical human studies examining the physiological, behavioral, cellular, and molecular effects of tryptophan metabolites in this context; and discuss the roles of tryptophan metabolites and the sex hormone estrogen in signaling within the brain–gut–microbiome axis under health and disease conditions. We carefully specify the animal models used and species tested in animal studies. When referring to animal studies, animal behavior is referred to as “anxiety-like” and “depression-like”, as animal models do not recapitulate the myriad of symptoms displayed by humans. It is worth noting that this review is unique compared to most of the available literature in two aspects. This review (1) offers a holistic view tying gut microbiome-related metabolic regulation to nervous system-related behavioral regulation and (2) summarizes sex-specific regulation and sex differences in behavior involving tryptophan and 5-HT in health and disease conditions. These insights may offer novel approaches to enhance brain–gut communication and improve metabolic outcomes in conditions such as eating disorders and mood disorders.

## 2. Tryptophan Metabolism in the Brain–Gut–Microbiome Axis

### 2.1. Overview of Tryptophan and Tryptophan Metabolism

Tryptophan, an aromatic amino acid, contains an indolic group attached to an alanyl side chain. In nature, it is produced by bacteria and plants through the shikimic acid and anthranilate pathways, which are utilized for the production of various medicinal indolic products [76]. In contrast, animals, including humans, cannot synthesize tryptophan; it is one of the eight essential amino acids that must be obtained from dietary sources [77]. Although certain saprophytic microflora, such as *Escherichia coli*, can produce tryptophan, they do not contribute significantly to human tryptophan levels [78]. Consequently, dietary intake is crucial, with sources including milk derivatives, chocolate, cereals, red meat, poultry, eggs, fish, and dried fruits, along with limited amounts from endogenous protein degradation [77].

Tryptophan is an essential amino acid, not only crucial for protein synthesis but also a substrate for several important bioactive compounds. Besides being converted to tryptamine, it is primarily metabolized through three pathways: (i) conversion to serotonin (5-hydroxytryptamine, 5-HT) and melatonin; (ii) conversion to kynurenine; and (iii) conversion to indole acetic acid. These metabolic pathways occur in both the CNS and the peripheral ENS, where tryptophan metabolites significantly influence a variety of metabolic and psychiatric functions critical for health [1]. Notable associations between tryptophan metabolites and various diseases are extensively documented in the literature. This review focuses specifically on 5-HT, one of the tryptophan metabolites and a key CNS neurotransmitter vital for regulating many physiological processes, including mood, appetite, and energy metabolism. While a detailed examination of all tryptophan metabolites is beyond the scope of this review, it is important to note that metabolites from the kynurenine and indoleacetic acid pathways are also recognized as significant metabolic regulators involved in inflammation and the immune response.

### 2.2. Tryptophan Metabolism Within the Microbiota–Gut–Brain Axis

#### 2.2.1. Various Tryptophan Metabolites Synthesized from Multiple Pathways

The biochemical cascades for tryptophan metabolism and tryptophan metabolite synthesis are intricately linked to the microbiota–gut–brain axis, with microbial regulation playing a significant role [79]. Dietary tryptophan, once absorbed through the intestinal epithelium, exists in both free and albumin-bound forms, either remaining in the gut or entering the bloodstream [78]. In the blood, tryptophan levels are influenced by dietary protein intake [80]. The free form of circulating tryptophan serves as the primary substrate for protein synthesis [77].

In the gut lumen, five microbial phyla—Actinobacteria, Firmicutes, Bacteroidetes, Proteobacteria, and Fusobacteria—metabolize tryptophan through various metabolic pathways [81,82]. Tryptophan transporters facilitate its uptake from the gut lumen into bacterial cells, allowing bacteria to sequester it from the host. Most tryptophan metabolism by the microbiota occurs in the distal colon, where bacterial proteolytic activity is the highest [78,83]. Some metabolism by *Lactobacilli* also occurs in more rostral regions such as the stomach and ileum [82].

Bacteria produce a few neuroactive tryptophan metabolites [84,85]. Around 90% of tryptophan is metabolized and gives origin to kynurenine and its derivatives kynurenic acid and quinolinic acid. Only about 3% is hydroxylated to intermediate 5-hydroxytryptophan (5-HTP), by the rate-limiting enzyme tryptophan hydroxylase (TPH), which is then converted to 5-HT via amino acid decarboxylase. Other pathways yield tryptamine and various indole derivatives, including indole, indole acetic acid (IAA), indole propionic acid (IPA), indole lactic acid, indole acrylic acid, and skatole [85,86].

#### 2.2.2. Various Neuroactive Tryptophan Metabolites

It is noteworthy that, while this review emphasizes 5-HT, many other tryptophan metabolites produced by the gut microbiota and the CNS act as essential neuroactive molecules and participate in brain–gut communication to regulate energy metabolism, mood, and behavior in humans and lab animal models [1,78,79,82,83,87,88,89,90,91]. For example, the central administration of quinolinic acid, a metabolite from the kynurenine pathway, reduces grooming behavior and shortens the duration of social interaction in mice [91], suggesting anxiogenic effects of quinolinic acid. Findings from animal studies are supported by clinical research. In humans, depression, anxiety symptoms, and cognitive impairments in patients with schizophrenia are associated with the increased production of tryptophan metabolites [92]. Additionally, in women, depression and anxiety related to body image satisfaction are linked to the activation of tryptophan catabolism and CNS availability of quinolinic acid [93]. In both preclinical animal studies and clinical human studies, increased immune responses enhance tryptophan catabolism, resulting in decreased levels of tryptophan and 5-HT, while increasing the production of neurotoxic tryptophan catabolites such as kynurenine and quinolinic acid [94,95]. This shift can lead to heightened depression and anxiety behaviors in both humans and rodents [80,96,97].

#### 2.2.3. 5-HT Synthesis

5-HT is the most studied neuroactive molecule among all tryptophan metabolites synthesized in both the gut and the brain. Serotonergic neurotransmission also occurs in both the gut and the brain. The majority of 5-HT is produced from tryptophan by the microbiome in the enterochromaffin cells (a subtype of enteroendocrine cells) of the gut [98]. Fluctuations in local 5-HT concentration can influence enteric neural processes and subsequently relay signals along the brain–gut axis, affecting CNS neurotransmission, as has been tested in human biopsy transverse colons and in colons from laboratory rodent [41,99]. Some tryptophan crosses the blood–brain barrier via large amino acid transporters to synthesize 5-HT in the CNS [79]. The experimental manipulation of tryptophan levels modified 5-HT levels in both the gut and the CNS [79].

Two well-characterized homologous isoenzymes of TPH, TPH1 and TPH2, are primarily expressed in the gut and in the CNS, respectively [100]. In humans, circulating tryptophan is used to synthesize 5-HT in the CNS via TPH2 [101,102]. It is noteworthy that, while circulating tryptophan concentration influences CNS 5-HT levels, it is not the sole factor. In clinical studies, other factors have been identified in humans. For example, competition with other amino acids for transport across the blood–brain barrier also plays a critical role [103], and the ratio of tryptophan to other amino acids also determines CNS 5-HT levels [80]. Physiological factors such as exercise can further influence tryptophan distribution and utilization in the CNS in humans [104].

#### 2.2.4. 5-HT Synthesis in the Gut by Gut Microbiota

Research supports the importance of gut microbiota in 5-HT synthesis. In humans, approximately 95% of the body’s 5-HT is secreted by mucosal enterochromaffin cells, regulated by microbiota. Certain gut bacteria, including *Enterococcus*, *Pseudomonas*, *Streptococcus*, and *Escherichia*, can produce 5-HT in tryptophan-rich media [11,86,105,106] by controlling amine transport, metabolism, and biosynthesis [107,108,109]. Additionally, short-chain fatty acids (SCFAs) and particularly butyrate, a metabolite produced by gut microbiota, have complex effects on 5-HT synthesis from human intestinal enterochromaffin cells. Low levels of butyrate promote TPH1 expression and increase 5-HT synthesis, while high levels of butyrate suppress TPH1 expression and decrease 5-HT synthesis [107].

Animal studies also indicate that gut microbiota significantly modulate 5-HT synthesis. Germ-free mice exhibit significantly lower 5-HT levels compared to conventionally housed mice, while the fecal flora colonization of germ-free mice with specific pathogen-free mouse microbiota rapidly increased colonic 5-HT levels within days [108]. Similarly, germ-free mice with human gut microbiota colonization increase their colonic TPH1 mRNA and protein levels, which consequently increases colonic 5-HT levels [107]. Pathogenic bacteria such as *Escherichia coli*, *Vibrio cholerae*, and *Salmonella typhimurium* have been associated with upregulated 5-HT secretion into the lamina propria of the gut [110]. These studies suggest that gut microbiota promote 5-HT production in the gut lumen.

#### 2.2.5. 5-HT Synthesis in the Gut Regulated by Microbial Metabolites

Diet also influences both local gut processes and systemic effects through bacterial structural components and microbial metabolites [86]. The gut microbiome produces many bioactive compounds affecting host physiology, such as SCFAs such as acetate, propionate, and butyrate [111] as well as choline metabolites and lipids [25]. SCFAs can rapidly move from the colonic lumen into colonic epithelial cells and provide a preferred energy source for the epithelia [111]. Some gut bacterial metabolites regulate 5-HT synthesis, and some are substrates for 5-HT, which can further regulate gut functions. For instance, in both mice and humans, metabolites from *Clostridium* species can stimulate 5-HT synthesis and release from colonic enterochromaffin cells, enhancing gastrointestinal motility [112] and affecting neurotransmitter and hormone activities [113,114].

Research has shown that butyrate levels are lower in both male and female mice fed a high-fat diet (HFD) compared to those on a low-fat diet (LFD). Notably, female mice exhibit more modest decreases in butyrate levels, while male mice show more pronounced reductions [29]. Consequently, HFD-fed mice, particularly males, have higher levels of gut-synthesized 5-HT compared to their LFD-fed counterparts [29].

In humans, lipopolysaccharides (LPSs) stimulated the release of 5-HT from enterochromaffin cells isolated from patients with Crohn’s disease [115]. This regulation of 5-HT synthesis and release from intestinal enterochromaffin cells by gut microbial metabolites represents a form of local paracrine regulation. These metabolites activate receptors located either on afferent nerve terminals near the enterochromaffin cells, triggering enteric reflexes, or directly on the enterochromaffin cells themselves [41,116].

To summarize, microbial products play critical roles in maintaining gut homeostasis. They facilitate the secretion of various enteric neuropeptides and cytokines from mucosal enteroendocrine and immune cells [82]. Additionally, they regulate the biosynthesis and release of various neurotransmitters and neuromodulators by enterochromaffin cells [84,117]. The released enteric neuropeptides, cytokines, neurotransmitters, and neuromodulators, along with microbial metabolites, diffuse through the lamina propria, accessing local receptors or entering the bloodstream, to affect local ENS neurons and remotely signal CNS neurons via vagal innervation [118,119,120]. Ultimately, these interactions help to regulate gut and CNS functions, including appetite and satiety, nutrient metabolism, energy homeostasis, immune responses, mood, and behavior [3,7,9,11,69,121]. In contrast, an HFD impacts the synthesis of SCFAs, which can regulate TPH1 levels and thus affect 5-HT synthesis [29]. The resulting imbalance in SCFA production may lead to the dysregulation of gut–brain communication and mood disorders, highlighting the importance of diet in shaping gut microbiota and their implications for mental health.

### 2.3. Sex Differences and Roles of Estrogen in Tryptophan Metabolism

#### 2.3.1. Hormone Regulation of Tryptophan Metabolism

Research indicates significant sex differences in tryptophan metabolism, likely influenced by sex hormones. In women, reduced tryptophan and 5-HT synthesis, along with the increased production of neurotoxic and anxiogenic tryptophan metabolites, particularly via the kynurenine pathway, are associated with eating disorders and mood disorders, such as depression and anxiety. Women, regardless of ethnicity, generally have higher levels of free tryptophan but lower total tryptophan levels in plasma compared to men [122]. Additionally, the kynurenine pathway differs between sexes. For example, African and Hispanic American women exhibit lower levels of tryptophan metabolites than their male counterparts [122]. Ethnicity also plays a role in tryptophan metabolism, as Caucasian women, in particular, show the lowest levels of kynurenic acid, which potentially correlates with a lower incidence of schizophrenia in the Caucasian female population [122]. Furthermore, dietary factors that induce acute tryptophan depletion can trigger increased stress-induced anxiety responses in women, such as during a simulated public speaking test [123], suggesting that women may be more sensitive to diminished 5-HT neurotransmission than men. These findings highlight the potentially significant roles of both sex and ethnicity in the complex relationship between tryptophan metabolism and mental health.

Tryptophan metabolism is also regulated by stress and immune responses. For example, in men and women with similar post-traumatic stress disorder severity, the level of indole metabolite (indole-3- propionic acid) decreases in women, but not in men, possibly due to gut microbiota dysbiosis [21]. Other studies have reported that increased levels of gut bacteria-derived LPSs can activate the kynurenine pathway, leading to elevated levels of the neurotoxic quinolinic acid [124]. This pathway activation is more pronounced in women than in men following immune challenges [80], which drives tryptophan metabolism and conversion into the tryptophan metabolite kynurenine, leading to increased kynurenine levels and decreased CNS 5-HT synthesis [124].

Such changes are not only more pronounced in women than in men, but also exacerbated during pregnancy and the menstrual cycle and commonly observed in women during the perinatal period, including at the end of pregnancy, during early postpartum, and in the premenstrual phase. These changes are associated with the heightened symptoms of depression and anxiety observed in pregnant and parturient women [94,124,125,126]. The dynamic fluctuations in sex hormones during pregnancy and the puerperium may enhance kynurenine pathway activity, increasing the risk of anxiety and depression during these life stages. However, one clinical study did not find a correlation between increased kynurenine levels and prenatal or postpartum depression, or at the end of term pregnancy [126]. This discrepancy may be due to the complex interactions between immune responses, stress, and tryptophan metabolism, especially during life stages related to pregnancy and menstrual cycles with dynamic physiological and behavioral changes [126,127,128,129].

#### 2.3.2. Hormone Regulation of 5-HT Synthesis

Both the sex hormones estrogen and progesterone contribute to enhanced 5-HT synthesis by promoting the expression and activation of key enzymes, such as protein kinase A [130] and, subsequently, phosphorylated TPH [131]. Specifically, estradiol increases TPH mRNA and protein levels without affecting 5-HT content in mice, and progesterone may activate protein kinase A [132]. In guinea pigs, when treated with combined estrogen and progesterone, both TPH and 5-HT levels in the dorsal raphe nucleus (DRN) and the hypothalamus are increased [133]. In ovariectomized (OVX) rats, a rodent model of postmenopause, estradiol treatment alone, but not combined estrogen and progesterone treatment, increased TRH gene expression in the DRN [134]. It is possible that postmenopausal women with lower levels of estrogen could exhibit lower expression of the TPH gene and protein or reduced activation of TPH, leading to reduced 5-HT synthesis and 5-HT levels in the CNS, potentially increasing the risk for mood disorders and eating disorders in postmenopausal women.

Despite the association between sex hormone levels and mood and eating disorders, not all postmenopausal women experience these conditions. A study has reported that sex hormone levels are not significantly associated with eating disorders in either premenopausal or perimenopausal women [135]. It is possible that the activity and expression of TPH and subsequent 5-HT synthesis, along with the activity of CNS serotonergic neurons, may adjust in response to dynamic changes in sex hormones during peri- and postmenopause in some women. These animal and human studies suggest a complex interaction between sex hormones and tryptophan metabolism, which may underly the sex differences in anxiety, depression, and eating disorders. Understanding sex differences and the effects of sex hormones on tryptophan metabolism pathways, especially in women, is critical for treating and preventing mental and eating disorders.

### 2.4. Sex Differences and Roles of Estrogen in Gut Microbiota

#### 2.4.1. Sex Specific Alterations in Gut Microbiota in Response to Obesogenic Diet

Sex differences in gut microbiota composition have been well documented, with men generally exhibiting higher levels of the Bacteroides and Prevotella groups than women [136]. Gene expression contributes to diet-induced sex-specific weight gain and fat distribution in both humans and laboratory animal models [137,138]. Dietary factors, rather than obesity itself, appear to induce sex-specific changes in gut microbiota composition. For instance, in rodent models, feeding mice an obesogenic HFD rapidly alters their gut microbiota at both the phylum and family levels, as well as the metabolite production of gut microbiota, often preceding the onset of diet-induced obesity [29]. In both sexes of mice, the consumption of an obesogenic HFD reduces the abundance of beneficial bacterial groups, such as *Bifidobacteria* [29], while increasing the abundance of *Firmicutes*, a microbiota phylum associated with enhanced energy harvest from food. These changes in turn promote obesity development and metabolic dysregulation in the host. Additionally, it has been reported that, using genetic mouse models with enhanced inflammation and diminished colonic barriers, *Bifidobacteria* play a crucial role in regulating the expression of tight junction protein, reducing the pro-inflammatory cytokines in mucus, and maintaining the epithelial barrier [139]. The findings of preclinical and clinical studies have indicated that a reduction in beneficial flora such as *Bifidobacteria* is closely linked with the development of chronic inflammation, a widely acknowledged contributing factor for various diseases, including cancer [140]. Interestingly, sex-specific differences in gut microbiota composition are observed in rodent models of HFD-induced obesity. The increase in *Firmicutes* in mice is substantially slower in females than in males [29].

Host gut microbiota also influence chromatin changes, such as DNA methylation and histone acetylation in colon epithelial cells. These modifications can alter the host epigenome, prime enhancers, and activate genes in the colon epithelium involved in functional signaling pathways related to metabolic dysfunction and cancer development. For example, the transplantation of microbiota from obese mice into germ-free mice has been shown to replicate HFD-associated epigenetic changes [29], leading to Dnmt1-independent DNA methylation changes in epithelial cells in *Dnmt*-deficient mouse models [141], modifications in the chromatin status of intestinal intraepithelial lymphocytes in germ-free mice [142], and changed host tissue chromatin features, resulting in alterations in host gene expression and physiology in germ-free mice [143]. These studies suggest that epigenetic changes may contribute to certain host outcomes related to the microbiome. Along the same line, germ-free animals reintroduced with microbiota from obese donors exhibit epigenetic changes at cancer-associated loci, indicating a potential connection between obesity and an elevated risk of colorectal cancer through these epigenetic modifications [144]. The interplay between epigenetic changes, inflammation, and tumorigenesis is likely influenced by the metabolism of obesogenic diets and gut microbiota, at least in laboratory mouse models.

Additionally, both the gut microbiome and the epigenomic responses, including chromatin characteristics, histone methylation, and histone acetylation in the colon epithelium, of mice fed an obesogenic diet are sex-specific [29]. A study by Lee et al. has reported that HFD feeding induces inflammation and cell proliferation in colon mucosa in both young (6 weeks old) and old (2 years old) rats of both sexes, while also causing gut dysbiosis characterized by decreased gut microbiota species richness and an increase in the abundance of specific microbial species, such as *Clostridium lavalense* that produce carcinogenic compounds, and an increase in the *Firmicutes*/*Bacteroidetes* ratio in old rats [145]. Notably, sex differences in gut microbiome alterations due to HFD feeding have been reported. For example, the abundance ratios of *Akkermansia muciniphila* and *Desulfovibrio* increase in response to HFD feeding in young rats of both sexes and old female rats, but not in old males [145]. Additionally, HFD feeding elevates the concentration of myeloperoxidase, an enzyme that produces reactive oxidants leading to tissue damage and inflammation, in the colon mucosa of male but not female rats [145]. These findings suggest sex differences in the composition of the gut microbiota in responding to obesogenic diets.

The consequences of the changes in microbiota associated with obesity include a reorganization of the microbiome, which subsequently affects disease risk. Thus, obesogenic diets and obesity exert differential effects on gut microbiota composition, abundance ratios, and microbial metabolite production in young and old males and females in laboratory mouse and rat models. This contributes to age- and sex-related differences in the development of inflammation and colon tumorigenesis, consistent with clinical studies that indicate a higher frequency of metabolic disorders and colon cancer in men compared to women [146,147,148,149,150,151].

#### 2.4.2. Sex Specific Alterations in Gut Microbiota Due to Estrogens

Sex dimorphism has been identified as an important contributor to the distinct sex differences in the maturation and maintenance of the brain–gut–microbiome axis, contributing to differences in disease risk and resilience between males and females throughout their lifespans. The potential mechanisms underlying these sex differences in neural circuitry, which regulate feeding, metabolism, and mood, are only beginning to be understood and will continue to be explored. These mechanisms have largely been attributed to the neuroendocrine interactions in the brain–gut–microbiome axis involving sex hormones and neurotransmission, especially during developmental stages.

Steroid estrogens typically bind to and activate their cognate nuclear estrogen receptors (ERs) ERα and ERβ. Besides their well-known influences in developing and maintaining reproductive tissues and behaviors, estrogens also influence various non-reproductive targets, including gut microbiome and the CNS. For example, findings from preclinical and clinical studies suggest that estrogen and gut microbiota act together to regulate weight gain and lipid deposition [152], beneficially impacting on metabolic disorders such as obesity, diabetes, and cancer.

Lifestyle factors such as sedentary behavior, restricted mobility, and diets high in fat and sugar contribute to obesity development. Estrogens are essential for maintaining metabolic homeostasis in healthy premenopausal individuals. However, postmenopausal women, who experience decreased estrogen levels, often exhibit a reduced metabolic rate and become more susceptible to obesity development, fat deposition to the abdominal area, lipid disorder, cardiovascular diseases, and insulin resistance, all hallmark features of metabolic syndrome [137,153,154]. In humans, gut microbiota also play a significant role in the development and progression of various diseases, including non-alcoholic fatty liver disease [155] and colorectal cancer [156], suggesting a strong association between gut microbiota and risks for disease development and progression. Emerging evidence points to an interaction between estrogens and the microbiome that may enhance metabolic rate, lower body weight, and reduce adiposity, thereby decreasing the likelihood of metabolic diseases.

Importantly, genes for a variety of estrogen-metabolizing enzymes have been identified within gut microbiota in laboratory rodent models and humans [157], indicating that gut microbiota can metabolize dietary and endogenous estrogens and produce their metabolites that act as ER ligands. Among men, premenopausal women, and postmenopausal women, increased concentrations of estrogenic metabolites, along with reduced estrogen levels, positively correlate with increased microbial diversity, measured using fecal samples, and a reduced risk for breast cancer and metabolic diseases [158]. Thus, estrogenic metabolites may mediate host metabolism and alleviate symptoms associated with metabolic diseases and cancer. Although the underlying mechanisms remain incompletely understood, estrogen metabolism by the gut microbiota serves as a critical biomarker for evaluating host health and disease risk. The replacement of both estrogen and probiotics may synergistically improve gut microbiota composition and mitigate the chronic inflammation seen in metabolic diseases, potentially preventing fatty liver disease and cancer more effectively.

## 3. Functional Roles of Tryptophan Metabolites in the Brain–Gut–Microbiome Axis

Tryptophan metabolism plays a key role in modulating host health and disease states through its metabolites, especially in the brain–gut–microbiome axis [82]. A wealth of evidence highlights the physiological significance of tryptophan metabolites in the interactions between the brain, gut microbiota, and enteric microenvironment. The enteric microenvironment includes various cell types, such as enterocytes, smooth muscle cells, enteroendocrine cells, neurons, glial cells, and immune cells, all of which communicate with the gut microbiota by both receiving and sending signals. Sex hormones may influence the regulation of tryptophan metabolic pathways, adding another layer of complexity to these interactions. This crosstalk is critical in maintaining bidirectional communication along the brain–gut–microbiome axis. Metabolites from tryptophan metabolism, particularly 5-HT, play vital roles in cognition, memory, and emotions in both animals and humans [159,160,161,162,163] and are closely associated with emotional and mood disorders (such as anxiety, depression, mood swings, and irritability) and feeding behaviors (such as food cravings), as well as cognitive functions and memory during the perinatal and premenstrual periods in humans, when sex hormone levels fluctuate and change dynamically [164].

### 3.1. Brain Tryptophan Metabolites Regulate Physiology and Behavior

The CNS and gut microbiota interact to influence a variety of physiological and behavioral processes within the brain–gut–microbiome axis. Among these processes, serotonergic neurotransmission is the most well-studied. 5-HT is central to regulating energy metabolism, including feeding behavior and metabolism [165], as well as mood disorders such as depression and anxiety in humans and depression-like and anxiety-like behavior in laboratory animals [166]. Different subtypes of serotonin receptors interact with 5-HT and are involved in these processes. Disruptions in serotonergic transmission can affect the functioning of CNS and contribute to the development of major depression and schizophrenia and other psychiatric disorders [36]. Additionally, preclinical and clinical studies have reported that changes in circulating tryptophan levels, influenced by the gut microbiota, can affect serotonergic neurotransmission, altering the function of both the CNS and ENS [36]. For instance, in a transgenic mouse model of autism spectrum disorders, reduced 5-HT levels in the gut correlate with an increased presence of bile-metabolizing bacteria like *Bifidobacterium* and *Blautia* [167]. Therefore, either an abnormal circulating level of tryptophan or changed 5-HT receptor expression may affect the functions of the brain–gut–microbiome axis.

#### 3.1.1. Brain 5-HT and Its Receptors and Transporter

While the mechanisms by which 5-HT regulates energy metabolism, feeding behavior, and mood remain largely unclear, it is evident that studying sex differences in 5-HT neurotransmission is an area of active investigation, with results often inconclusive. Within the CNS, 5-HT is known to modulate mood, behavior, and cognitive functions. Low 5-HT levels are associated with depression, fatigue, and impaired cognition [168]. Serotonergic drugs, such as selective serotonin reuptake inhibitors (SSRIs), which interfere with the 5-HT transporter to increase the amount of 5-HT at the synaptic junctions among neurons, are commonly used to treat anxiety and depression by increasing synaptic 5-HT levels [169]. Furthermore, reduced levels of circulating tryptophan are linked to mood disorders, which can often be successfully treated with SSRIs [170].

Most 5-HT-expressing neurons project to the prefrontal cortex and hippocampus to integrate cognition and memory processes, to the hypothalamus to regulate energy homeostasis, and to the limbic system to mediate arousal and regulate mood. 5-HT exerts its effects in both the gut and the CNS through a variety of 5-HT receptors, which are divided into seven families (5-HT1 to 5-HT7). Except for the 5-HT3 receptor, which is a ligand-gated ion channel for Na^+^ and K^+^, the rest of the 5-HT receptors are G protein-coupled receptors [171]. While most 5-HT receptors are expressed in the CNS, 5-HT3 and 5-HT4 receptors are primarily found in the gastrointestinal tract. The key receptors involved in regulating anxiety include 5-HT1A, 5-HT2A, 5-HT2C, and 5-HT3. Notably, sex differences have been observed in the expression and function of these receptors, particularly in the context of depression and anxiety. In mammalians, including human and rodents, the serotonin transporter (SERT) plays a critical role in 5-HT signaling by moving 5-HT from the synaptic cleft into presynaptic neurons [172] where 5-HT is degraded. Thus, SERT reduces 5-HT content at the synaptic cleft and terminates 5-HT neurotransmission.

#### 3.1.2. Sex Differences and Estrogen Modulation in Brain Serotonin Neurotransmission

Estrogen influences 5-HT function through both genomic and non-genomic pathways, affecting the expression and activity of various serotonin receptors and SERT. In the CNS, the majority of 5-HT is synthesized in neurons located in the DRN of the midbrain. Both ERα and ERβ are expressed in serotoninergic neurons, particularly in the brain regions of the hypothalamus and DPN. ERα is expressed in the DRN of mice [132], while ERβ is expressed in the DRN of guinea pigs [133] and nonhuman primates [173,174]. Estrogen can regulate 5-HT neurotransmission through the activation of these receptors. For example, estradiol treatment has been shown to enhance 5-HT neuronal activity in the DRN of male and female rats [175,176]. Additionally, the selective activation of ERα using the agonist propylpyrazole-triol (PPT) increases 5-HT neuronal activity in the DRN of mice [132]. This activation is blocked when ERα is genetically deleted from the DRN’s serotonergic neurons [132], confirming that the effects of PPT on 5-HT neurons are dependent on ERα. Furthermore, the selective activation of ERβ tends to suppress, while activation of ERα has the opposite effect and induces, depression- and anxiety-like behaviors in rodent models [177,178,179].

##### 5-HT1A Receptor

One of the most studied 5-HT receptors in relation to estrogen, the 5-HT1A receptor, is primarily expressed in the hippocampus, cortex, septum, median raphe nucleus, and other brain regions [180,181]. Preclinical animal studies and clinical studies have shown that the 5-HT1A receptor is both an autoreceptor located on presynaptic neurons and a postsynaptic receptor [180,181]. For example, in laboratory rats, the activation of 5-HT1A receptor inhibits serotonergic neuron firing, reduces 5-HT synthesis [182], and suppresses 5-HT release [183], thus decreasing the 5-HT amount present in the synaptic cleft in the hippocampus [184,185].

The sex hormone estradiol has been shown to modulate the expression of the 5-HT1A receptor, with suppression observed in the dentate gyrus and CA2 of the hippocampus as well as in the DRN of OVX rats [186] and in the dorsal and median raphe nuclei of OVX rhesus monkeys [187]. This regulation of 5-HT autoinhibition by estrogen is important for behaviors such as eating, mood, and metabolism. For instance, estrogen administration has been found to decrease the expression and binding of the 5-HT1A receptor in rats [188]. In rat models, 5-HT1A autoreceptor agonists, such as 8-OH-DPAT, reduce 5-HT release from the DRN, resulting in increased eating behavior and food intake [189,190]. Additionally, estrogen’s influence on feeding behaviors can counteract the anorectic effects of SSRIs. Sex differences and estrogen’s effects are observed in this 8-OH-DPAT activation of the 5-HT1A autoreceptor-induced feeding stimulation, counteracting SSRIs’ anorectic effect. Specifically, the 8-OH-DPAT-induced reversal of SSRI-induced feeding suppression is more effective in male rats and in female OVX rats compared to intact females [190]. Moreover, this effect is more pronounced during the diestrus phase than during proestrus or estrus [190], and in control-treated rats than in estrogen-replaced OVX rats [191] when estrogen levels are higher. Thus, reversing the SSRI-induced feeding suppression by 5-HT1A autoreceptor activation is more effective in males and when estrogen levels are low, suggesting that estrogen enhances 5-HT’s effects on feeding suppression by downregulating 5-HT1A autoreceptor activity or expression. Therefore, activating the 5-HT1A autoreceptor with agonists such as 8-OH-DPAT increases feeding behavior in animal studies, and this effect is more pronounced in male rats and in OVX female rats with low estrogen levels [188,190,191].

##### 5-HT2A Receptor

Postsynaptic 5-HT2A receptors are predominantly located in the cortex, ventral striatum, hippocampus, amygdala, caudate nucleus, nucleus accumbens, and olfactory tubercle [181,192]. Animal studies have suggested that these 5-HT2A receptors could receive signals from the DRN and play a key role in regulating anxiety-related behavior, with the disruption of 5-HT2A receptor signaling in 5-HT2A receptor knockout mice leading to increased anxiety-like behavior, a response that could be reversed by restoring proper 5-HT2A receptor function [192].

##### 5-HT2C Receptor

The postsynaptic 5-HT2C receptor is expressed in several brain regions, including the dorsal striatum—where the DRN projects to—cortex, nucleus accumbens, hippocampus, amygdala, and basal ganglia [181]. In laboratory rats, the activation of the 5-HT2C receptor in the basolateral amygdala induces fear-like behavior and impairs stress-related learning [193,194]. Mice with an overexpression of the 5-HT2C receptor exhibit increased anxiety-like behavior [195], while mice with 5-HT2C receptor absence show reduced responses to anxiety stimuli and suppressed anxiety-induced activation of corticotropin-releasing hormone (CRH) neurons [196]. Notably, the gene encoding the 5-HT2C receptor is located on the X chromosome in both humans and rodents [181,197], which could contribute to the sex differences in stress-induced anxiety. Females, with two X chromosomes, may be more prone to anxiety due to a higher expression of the 5-HT2C receptor compared to males, who have only one X chromosome.

Estrogen modulates serotonergic regulation in feeding and satiety via regulating the expression of the 5-HT2C receptor [198,199,200]. Interestingly, estradiol treatment suppresses 5-HT2C receptor expression in the hypothalamus of OVX macaques [201], but elevates its expression in the caudal brainstem of OVX rats [202]. Therefore, the effects of estrogen on the 5-HT2C receptor are brain region-specific.

These animal studies suggest a potential mechanism through the 5-HT2C receptor to affect anxiety-like and feeding-related behaviors and contribute to the understanding of the neural circuitry that mediates anxiety and feeding in men and women.

##### 5-HT3 Receptor

Different from other 5-HT receptors, which are G protein-coupled receptors, the 5-HT3 receptor is a ligand-gated ion channel on both presynaptic and postsynaptic neurons. It is primarily expressed in the hippocampus, amygdala, and cerebral cortex [181]. In rats, the activation of the 5-HT3 receptor in the amygdala by its agonist induces avoidance-related behavior, while inhibition of the receptor by its antagonist suppresses this behavior [203]. Interestingly, sex differences in anxiety-like behaviors are also observed with 5-HT3 receptor deficiency. Female 5-HT3 receptor-deficient mice spend more time in the open field, whereas male mice with 5-HT3 receptor deficiency spend less time, compared to their wildtype same sex counterparts [204], suggesting that 5-HT3 receptor deficiency reduces anxiety-related behavior in females but increases it in males.

##### SERT

SSRIs exert their anorexigenic effects by inhibiting SERT, thereby reducing the reuptake of 5-HT and increasing its concentration in the synaptic cleft, consequently suppressing feeding. In rats, females have higher levels of 5-HT in the CNS than males [205]. Estrogen has been shown to modulate SERT expression, as evidenced by a decrease in SERT mRNA levels in the DRN of OVX rhesus monkeys treated with estrogen [206]. Estrogen treatment also reduces [^3^H]paroxetine binding, a selective indicator of 5HT reuptake, in the hippocampus of rats [207]. These findings suggest the possibility that postmenopausal women receiving estrogen replacement therapy may exhibit decreased gene and protein expression of SERT, which helps to maintain higher levels of 5-HT in the synaptic cleft, supporting prolonged 5-HT neurotransmission and feeding suppression.

### 3.2. Gut Microbial Tryptophan Metabolites Regulate Gut Function and Metabolism

The gut microbiota produces a variety of components that play crucial roles in physiological processes, including LPSs, SCFAs, polyamines (e.g., putrescine, spermidine, spermine), bile salts (produced by the liver and modified in the gut lumen by the microbiota), and certain essential vitamins, as well as gas, toxins, and virulence factors, as has been shown in both preclinical and clinical studies [208,209,210]. Additionally, some metabolites derived from amino acids, including tryptophan metabolites (e.g., 5-HT, kynurenines, tryptamine, and indolic compounds) [81], tyrosine metabolites (e.g., dopamine, norepinephrine, and epinephrine), and glutamate metabolites (e.g., gamma-aminobutyric acid [GABA] and glutamic acid) [78,83,211], are particularly significant in regulating various physiological functions.

Recent evidence from animal studies, particularly antibiotic treatment-induced dysbiosis in mice, suggests that the gut microbiota can influence the structure and function of the ENS through both direct and indirect interactions with enteric neurons and glial cells [212,213]. In juvenile mice, chronic antibiotic treatment, which induces dysbiosis, leads to significant changes within the ENS. These include disruptions to the enteric glial network, changes in both excitatory and inhibitory motor neurotransmission, and an increase in neurotrophic signaling, such as brain-derived neurotrophic factor and its high-affinity tropomyosin-related kinase B receptor, in sensory and motor myenteric neurons [212,213].

#### 3.2.1. 5-HT Regulates Gut Functions

In the gut, 5-HT involves hormonal and neuronal actions to regulate various critical physiological functions, including mucosal secretion via regulating releases of Cl^−^ and water into the lumen, nutrient absorption, vasodilation, gastrointestinal tract motor and sensory functions, as well as pain perception [41,99,214,215].

The neuronal action is highly conserved across various animal species including fish and mammals and can be detected by the cation current and potential related to depolarization and hyperpolarization, as well as by the expression of several neurotransmitters including substance P, acetylcholine, calcitonin gene-related peptide, and 5-HT [212,216]. In mammals, 5-HT in the ENS is released from EC cells, where it stimulates intrinsic afferent neurons that project to the mucosa, thus promoting the activation of enteric reflexes [217]. These reflexes help to regulate smooth muscle contractions and motility in the gastrointestinal tract.

The release of 5-HT is modulated by both chemical and mechanical stimuli in the mucosa, which are relayed through intrinsic afferent neurons to both descending and ascending interneurons, as well as to excitatory and inhibitory motor neurons that innervate and control the smooth muscle layers of the gut. 5-HT also acts as a neurotransmitter released from myenteric neurons involved in the peristaltic reflex neural pathway [217]. While these findings highlight 5-HT as a key mediator of gut motility, it worth noting that the depletion of endogenous 5-HT does not completely block peristalsis in the large intestine or impede gut transit in vertebrates [218].

When germ-free mice are colonized with microbiota from conventionally raised mice, observable changes occur in serotoninergic pathways, leading to increased gut motility. These changes are driven by 5-HT release from both EC cells and enteric neurons, and are associated with the proliferation of enteric neuron progenitors in the gut of adult mice [219]. This underscores the importance of 5-HT in ENS neurogenesis and neuroprotection [36,41,219].

#### 3.2.2. Tryptamine Regulates Gut Functions

Tryptamine, another tryptophan metabolite, may indirectly enhance gastrointestinal motility by triggering the release of 5-HT from the EC cells of guinea pigs [220]. It has been reported that, in in vitro mouse colonic epithelial cells, tryptamine directly affects ion secretion, food particle transition, and bacterial cell movement through the gut lumen [84]. This suggests that tryptamine plays a role in modulating both intestinal motility and gut microbiota interactions.

In addition to influencing the structure and function of the ENS through EC cells, the gut microbiota, along with its metabolites, also directly impacts intrinsic afferent neurons [43,221]. Research in germ-free mice has revealed reduced excitability in myenteric intrinsic afferent neurons, a condition that can be restored when the mice are introduced to a normal gut microbiota [222]. For example, *Lactobacillus reuteri* has been shown to increase neuronal excitability by enhancing the number of action potentials per depolarizing pulse, reducing calcium and potassium ion channel opening and decreasing slow after-hyperpolarization in intrinsic afferent neurons [43]. Specific bacterial strains also contribute to normal intestinal motor function. In germ-free rats, colonization with *Lactobacillus acidophilus* or *Bifidobacterium bifidum* can partially reverse neuromuscular dysfunction and delayed intestinal transit, while colonization with *Escherichia coli* or *Micrococcus luteus* can delay gut motility [223].

In summary, gut microbial metabolites, particularly tryptophan-derived compounds such as 5-HT and tryptamine play an essential role in regulating gut motility and energy metabolism. The microbiota influences the ENS through direct interactions with neurons and glial cells, and indirectly by modulating neurotransmitter release and neuronal excitability. Convincing evidence from the colonization of germ-free rodent models supports these results. These findings highlight the significant impact of the gut microbiome on gut function, neurogenesis, and overall energy homeostasis (Table 1).

### 3.3. Diseases Related to Tryptophan Metabolism and 5-HT Neurotransmission in the Brain–Gut–Microbiome Axis

Certain gut microbiota, including *Bifidobacterium infantis, Bifidobacterium, Candida, Enterococcus*, *Escherichia coli, Lactobacillus acidophilus,* and Streptococcus synthesize and secrete a variety of neurotransmitters and neuromodulators, such as acetylcholine and 5-HT, which regulate memory, attention, learning, mood, and motivation, thus offering therapeutic effects for certain mental illness. For example, *Escherichia, Candida, Streptococcus*, and *Enterococcus* produce 5-HT; *Lactobacillus* species produce acetylcholine; *Escherichia coli*, *Bacilli*, and *Serratia* produce dopamine; *Candida, Streptococcus, Lactobacillus*, and *Bifidobacterium* species produce GABA; *Candida* and Streptococcus produce glycine; and *Escherichia* and *Bacilli* produce norepinephrine [11,86,225,226]. Neuroactive molecules secreted by intestinal microbiota can regulate nerve signals, influencing behaviors such as sleep, appetite, and cognitive behavior, as well as neuropsychiatric responses involved in stress responses and mood regulation in humans [226,227]. Thus, microbiota can interact with these neurotransmitters and neuromodulators to impact many aspects of physiology and behavior.

#### 3.3.1. Brain–Gut–Microbiome Axis Dysfunction in Eating Disorders

In some cases, changes in neurotransmitter levels during stress may be attributed not to the stress itself, but to the gut microbiota. For example, Crumeyrollearias et al. have reported that in germ-free rats, concentrations of homovanillic acid (HVA) decrease in the anterior cortex, hippocampus, and striatum, leading to a reduced dopaminergic conversion rate and a lower HVA/dopamine ratio [228]. This change is unrelated to this open-field stressor, as the effects of this stressor show similar concentrations of norepinephrine, 5-HT, and its metabolite 5-hydroxyindole acetic acid (5-HIAA) in the anterior cortex and hippocampus, whether in germ-free or specific pathogen-free rats under stress. The reduced dopaminergic conversion rate in germ-free rats may result from a reduction in the expression or activity of DA degradation enzymes, such as catechol-O-methyltransferase and monoamine oxidase (MAO) [228]. Depression has been associated with a downregulated tryptophan metabolism, as evidenced by an increased plasma kynurenine/tryptophan ratio. Kelly et al. have demonstrated that germ-free rats administered microbiota from depressed patients also show an elevated HVA/dopamine ratio [229].

Animal studies have shown that the 5-HT neurotransmission system plays a crucial role in regulating feeding behavior [161]. The dysregulation of 5-HT neurotransmission not only affects cognition and memory but also disrupts energy metabolism and emotional responses related to feeding [162,163]. Enhanced 5-HT neurotransmission, as observed with drugs like SSRIs that block SERT and elevate synaptic 5-HT levels [169], generally suppresses eating in animals [200]. SSRIs also interact with other neurohormones that regulate feeding. For example, enhanced 5-HT neurotransmission through treatment with fluoxetine, an SSRI, reduces the expression of the orexigenic neuropeptide Y in the hypothalamus and suppresses feeding in rats [230]. The interaction between 5-HT and neuropeptide Y is species-dependent, as fluoxetine treatment simultaneously elevates the gene expression of both orexigenic neuropeptide Y and anorectic corticotropin-releasing factor 1 (CRF1) in the hypothalamus of female goldfish, leading to weight loss [231].

Interestingly, the suppression of eating due to enhanced 5-HT signaling through SERT inactivation by the SSRI fenfluramine is more pronounced in female rats than males, and more evident in the estrus than in the diestrus phase during the estrous cycle of female rats [232]. This suggests that sex differences in feeding suppression by 5-HT exist, potentially enhanced by estrogen. When estrous cycle phases are not considered, no sex differences in feeding responses to fluoxetine are observed [190]. It is noteworthy that the kinetics and metabolism of different SSRIs vary. For example, fluoxetine is metabolized more quickly in female mice than in males [233]. Additionally, it is possible that sex differences in the feeding response to SSRIs may be more apparent between estrous females and males if estrous cycles are considered.

Besides suppressing feeding and inducing satiation, the 5-HT system also regulates nutrient selection. For instance, in macronutrient selection tests in rats, enhanced 5-HT signaling through the administration of 5-HT [234,235], fluoxetine (an SSRI) [236], or 5-HT agonists [237], either systemically or via intra-hypothalamic administration, suppressed carbohydrate intake in male rats without affecting fat or protein consumption. In contrast to enhanced 5-HT signaling by 5-HT, SSRIs, or 5-HT agonists, 5-HT receptor antagonists enhance carbohydrate consumption and reduce carbohydrate satiation in rats [238]. These studies suggest that, in male rats, macronutrient selection regulated by 5-HT signaling is carbohydrate-specific, without affecting protein or fat consumption. In female rats, however, enhanced 5-HT signaling via the administration of the SSRI fluoxetine suppresses the consumption of fat and protein, but does not impact carbohydrate intake [239]. Thus, while macronutrient selection regulated by 5-HT signaling appears carbohydrate-specific in males, this is not the case in females.

#### 3.3.2. Brain–Gut–Microbiome Axis Dysfunction in Anxiety and Depression

Numerous studies have shown that gut microbiota have beneficial effects on behavior. For instance, *Lactobacillus* and *Bifidobacterium* species reduce anxiety-like and depression-like behaviors, alleviate mood disorders, and prevent stress-induced changes in colonic microbiota [240,241,242].

Early life is a critical period for the development of the CNS, including the hippocampus, and for the establishment of neurotransmission systems such as the serotonergic system. Clarke et al. have reported sex differences in the relationship between anxiety-like behavior and CNS 5-HT [70]. They have found that germ-free rats, particularly male rats, exhibit increased circulating tryptophan (a 5-HT precursor) and elevated hippocampal levels of 5-HT and a main 5-HT metabolite 5-HIAA, compared to conventionally colonized rats. In contrast, female germ-free rats do not show any changes in these parameters [70].

These findings suggest that gut microbiota regulate circulating tryptophan, influencing CNS 5-HT levels and 5-HT neurotransmission through a humoral route. The differences in CNS 5-HT levels between males and females may be influenced by the estrous cycle and sex hormones. The restoration of normal gut microbiota through transplantation or probiotic administration normalized circulating tryptophan levels and alleviated anxiety-like behavior in germ-free rats, but did not reverse CNS 5-HT levels [70]. The study suggests that gut microbiota regulates both CNS 5-HT neurotransmission and anxiety-like behavior, both of which are disturbed by the absence of normal gut microbiota. The resistant 5-HT levels indicate that CNS neurobiochemical and 5-HT neurotransmission changes caused by a lack of microbiota in early life are not easily reversed. Something during CNS development may have a stronger effect on CNS 5-HT neurotransmission than the normalized microbiota. These findings highlight the complexity of the relationship between CNS 5-HT and anxiety-like behavior, as restored microbiota do not fully reverse the biochemical changes in 5-HT neurotransmission caused by early-life microbiota disruption, and recovered anxiety-like behavior cannot be explained by still-elevated CNS 5-HT levels. We have reported that, in a rat study, while the changes in relative microbial abundance caused by adolescent chronic stress faded three weeks after the stress ended in young adults, changes in microbial metabolic profiles persisted into adulthood in male rats [243]. Therefore, metabolites associated with altered gut microbes may offer therapeutic potential for stress-related disorders.

5-HT is one of the most important monoamine neurotransmitters that regulates the development of the immature mammalian brain during the neonatal stages, and 5-HT neurotransmission continues to regulate anxiety throughout life. The effects of 5-HT on anxiety have also been studied in the prenatal context. Changes in endogenous 5-HT levels, influenced by MAO activity, can profoundly impact brain development and anxiety responses. In mice, sex differences in 5-HT deficits during the prenatal stage have been observed, with female mice showing reduced anxiety-like behavior in the elevated plus-maze and dark–light chamber tests, while male mice exhibit heightened anxiety-like behavior [244]. This underscores the differential effects of 5-HT on males and females, even during prenatal development. The study of sex differences in 5-HT-related anxiety could improve personalized treatment approaches for anxiety. A better understanding of how anxiety manifests in pregnant females or women of childbearing age would facilitate more effective treatments, thereby reducing its impact on developing infants.

Sex differences in stress responses are evident in studies of the 5-HT system at the hippocampus and midbrain. The cooperation between 5-HT and the HPA axis increases in response to stress, especially in females. In rats, females show increased binding at 5-HT1A receptors in regions such as the Cornu Ammonis (CA) 4 and CA1 of the hippocampus in response to stress, independent of estrous cycle phase [245]. Stress-exposed female rats also exhibit higher corticosterone levels, with a more pronounced decrease over time than males [245]. The administration of a 5-HT1A receptor antagonist increases neuronal activity in the midbrain DRN serotonergic neurons in both male and female rats, but decreases corticosterone levels only in male rats, not in female rats [246].

Both the HPA axis and central 5-HT neurotransmission, modulated by CRH, are necessary to modulate stress-induced adaptive coping behavior. The dysregulation of these systems increases susceptibility to affective disorders like depression and anxiety. Sex differences in the regulation of the HPA axis via 5-HT activity contribute to the higher incidence of stress-induced depression and anxiety in women compared to men. For example, in humans, men show a greater cortisol response to the 5-HT precursor 5-HTP than women, but in patients with affective disorders, this relationship is reversed, with women exhibiting heightened cortisol responses to 5-HTP [247]. Studies in genetically stress-sensitive mice (e.g., CRH receptor 2-deficient mice) further support the idea that increased 5-HT levels via SSRI administration can override dysfunctional HPA axis regulation and reduce anxiety [248]. These findings suggest that stress-induced sensitivity to 5-HT, particularly in females, may underlie their increased vulnerability to stress-related affective disorder.

Interestingly, SSRIs have distinct sex effects in humans, with women being less inhibited by SSRIs in 5-HT reuptake than men. Additionally, the effect of SSRIs in women depends on the different follicular, ovulatory, and luteal phases of the reproductive cycle (also known as the endometrial and menstrual cycles) [249,250], with greater 5-HT reuptake inhibition at the beginning of menstruation when estrogen and progesterone levels are low in women.

Therefore, the cooperation between the central regulation of the HPA axis and 5-HT neurotransmission is sex-dependent, with women having greater responsivity, which may contribute to the higher incidence of stress-induced depression and anxiety in women compared to men.

## 4. Conclusions

Eating disorders and mood-related affective diseases, such as anxiety and depression, are significant global health problems. This highlights the urgent need to elucidate the underlying etiology of these diseases and develop targeted therapies or preventative measures. However, due to the complex interplay between host genetics, environmental factors, and the symbiotic gut microbiota, identifying primary causes remains challenging. Increasing evidence suggests that the dysbiosis of the gut microbiota plays a role in the pathophysiology of obesity, eating disorders, and stress [30,226,227,243]. Recent studies are beginning to clarify the molecular mechanisms through which intestinal bacteria can influence host physiology in profound ways.

In this review, we focus on available preclinical and clinical research regarding the regulation of metabolism and the nervous system by tryptophan metabolites, particularly 5-HT. We explore how the brain–gut–microbiome axis links physiological processes and behavior, and how disruptions in this axis contribute to metabolic and mental diseases. A key aspect of this discussion is the influence of sex differences and estrogen on tryptophan metabolism and 5-HT-related functions. It is crucial to understand how tryptophan metabolites, including 5-HT, affect both metabolic regulation and behavior (e.g., depression and anxiety), with significant variations across sex, estrous cycle phases, ovarian hormone levels, and reproductive stages such as puberty and pregnancy.

Sex-specific differences in the effects of tryptophan and 5-HT on behavior and metabolism are well-documented, as both preclinical and clinical studies have demonstrated that these effects are influenced by factors such as the estrous cycle, ovariectomy, and ovarian hormone fluctuations. Understanding these differences is essential, especially when considering the potential therapeutic applications for conditions like depression and anxiety. This review also emphasizes the potential for targeting the gut microbiome to modulate the serotonergic system as a novel therapeutic strategy for these conditions.

The outcomes of this review highlight the importance for further investigation into the molecular mechanisms behind the crosstalk between the brain–gut–microbiome axis and neural and humoral signals, particularly how it affects tryptophan metabolism, 5-HT synthesis, and the production of neuroactive metabolites. We speculate that altering gut tryptophan and 5-HT levels may play a critical role in the pathophysiology of higher-order brain functions, suggesting the gut microbiota as a promising therapeutic target for metabolic disorders. Although animal models have offered valuable insights into disease pathogenesis, mechanisms, and potential therapies, there are still gaps that need to be addressed. Future research should aim to bridge human and animal studies to better understand the impacts of tryptophan metabolites on CNS neurotransmission and related mental disorders, as well as peripheral glucose and lipid metabolism in the context of metabolic disorders. This approach could facilitate the development of new therapeutic strategies targeting tryptophan metabolism, either through conventional pharmacological treatments or by modulating the microbiota with prebiotics and probiotics.

In conclusion, advancing research in this field offers the potential to develop novel therapeutic approaches targeting tryptophan-generated metabolic pathways that are not the same between men and women and are unique during different life stages. Such strategies could be crucial for treating diseases characterized by significant CNS involvement, such as eating disorders, anxiety, and depression, for both men and women. Moreover, understanding the pathogenesis of these diseases through the lens of tryptophan metabolism could lead to innovative therapeutic tools that manipulate the microbiome, offering new avenues for treatment.

## Figures and Tables

**Table 1:** Research evidence of sex differences and effects of sex hormones on tryptophan metabolism and serotonin-related neuropsychiatric disorders.

	Sex Differences	Sex Hormone Effects
**Systemic Tryptophan Metabolism**	Tryptophan: male > female [122] 5-HT synthesis: male > female Tryptophan metabolites: male < female [80,122,124]	Low estrogen level reduces 5-HT synthesis and 5-HT levels [130,131,132]. Estrogen increases 5-HT level [133].
**Brain 5-HT Metabolism**	Levels of 5-HT in the CNS: male < female [205] Activation of 5-HT1A autoreceptor by 8-OH-DPAT: male > female [188,190,191]	Estrogen suppresses 5-HT1A autoreceptor expression [186,187,188]. Estrogen activates 5-HT neurons [175,176]. Effects of estrogen on 5-HT2C receptor are brain region-specific [201,202]. Estrogen decreases SERT expression [207].
**5-HT-Related Neuropsychiatric Disorders**	Eating disorders: male < female Mood disorders: male < female Anxiety: male < female [123]	Eating disorders are high in pregnant and parturient women [94,124,125,126] and also high in some postmenopausal women [135,224].

## Data Availability

No new data were created or analyzed in this study.
